# The benefits of GLP1 receptors in cardiovascular diseases

**DOI:** 10.3389/fcdhc.2023.1293926

**Published:** 2023-12-08

**Authors:** Lamija Ferhatbegović, Denis Mršić, Amra Macić-Džanković

**Affiliations:** ^1^ Department of Internal Medicine, Cantonal Hospital Zenica, Zenica, Bosnia and Herzegovina; ^2^ Clinic for Internal Diseases, University Clinical Center Tuzla, Tuzla, Bosnia and Herzegovina; ^3^ Faculty of Health Studies, University Vitez, Vitez, Bosnia and Herzegovina

**Keywords:** glucagon-like peptide-1, glucagon-like peptide-1 receptor agonist, cardiovascular diseases, major adverse cardiovascular events, diabetes mellitus

## Abstract

Glucagon like peptide-1 (GLP-1) receptor agonists are well established drugs for the treatment of type 2 diabetes (T2D). In addition to glycemic control, GLP-1 receptor agonists have beneficial other effects. They act by binding to GLP-1 receptors, which are widely distributed in the body, including cardiomyocytes and blood vessels. The aim of this article is to provide a comprehensive review of GLP-1 receptor agonists impact on cardiovascular outcomes and risk reduction. In the last decade, several cardiovascular outcomes trials (CVOT) have been conducted in order to explore cardiovascular benefit of GLP-1 receptor agonists. CVOTs primarily proved cardiovascular safety and tolerability of different GLP-1 receptor agonists, but also showed cardiovascular benefit of specific drugs. CVOTs have shown that GLP-1 receptor agonists reduce MACE in patients with T2D compared to placebo. In addition, they have positive impact on several cardiovascular risk factors such as obesity by promoting weight loss, blood pressure and blood lipid levels. Also, they stimulate the endothelium to produce nitric oxide, reduce oxidative stress, and have antiatherogenic and antiinflammatory effects. Studies have shown their positive impact on kidney outcomes in patients with T2D compared to placebo. The results of previous trials are encouraging in terms of multiple positive effects of GLP-1 receptor agonists. However, further research is needed to understand their full potential and all details of their mechanism of action, which will enable to expand the therapeutic indications and to determine their optimal use in clinical practice.

## Introduction

Despite major advance in treatment, cardiovascular diseases are still one of the leading causes of mortality and morbidity in the world. Among patients with type 2 diabetes (T2D), the risk of CVD is substantially elevated (1). To address this critical issue, the past decade has witnessed a surge in cardiovascular outcome trials (CVOTs) focused on evaluating the cardiovascular safety and efficacy of various antidiabetic drugs. One class of drugs that has gained significant attention in this context is the glucagon-like peptide-1 (GLP-1) receptor agonists. CVOTs evaluating GLP-1 receptor agonist’s (GLP-1 RA) effects on cardiovascular outcomes have consistently shown a reduction in MACE rates, primarily in rates of non-fatal myocardial infarction, non-fatal stroke and cardiovascular death. A positive effect on cardiovascular outcomes is achieved through several mechanisms (2). GLP-1 RAs are associated with blood pressure reduction, lipid levels reduction, weight loss and endothelial function improvement (3–6). The aim of this article is to discuss CVOTs involving GLP-1 receptor agonists and their impact on cardiovascular risk.

## Glucagon-like pepetide-1

GLP-1 is an incretine hormone that has a pivotal role in metabolic processes. Primary site of GLP-1 secretion are L-cells in small intestine and colon, but it is also secreted by the pancreatic α cells and neurons in nucleus tractus solitarus in the brainstem. There are evidence that microglial cells also secrete GLP-1 in mice. GLP-1 secretion itself is stimulated in response to food intake (7–9).

GLP-1 was discovered in 1981 when it was isolated from the pancreatic islet of anglerfish. Further research identified GLP-1 in L cells of mammalian gut mucosa and later, it was proved that GLP-1 enhances insulin secretion in response to nutrient ingestion (7, 10, 11).

GLP-1 exerts physiological effects by binding to the GLP-1 receptor (GLP-1R) on target cells. The activation of GLP-1R triggers a complex intracellular signaling cascade, which at the end activates protein kinase A (PKA) pathway via production of cyclic adenosine monophosphate (cAMP) (12). GLP-1R are found, apart from the pancreas, in lungs, kidney, central nervous system, stomach, cardiomyocytes, and vascular endothelial cells (13).

GLP-1 exerts potent glucose-lowering effects through multiple mechanisms including insulin release stimulation and suppression of glucagon secretion. The risk of hypoglycemia is significantly reduced since these effects are in close relationship with glucose blood levels (14). Also, GLP-1 improves beta cell survival by promoting beta-cell proliferation and regeneration and inhibiting apoptosis (15).

GLP-1 is a pleiotropic hormone with numerous functions besides glucose control. These multiple GLP-1 effects are observed due to wide distribution of GLP-1 R. For example, different actions of GLP-1 receptor agonists such as delaying gastric emptying which leads to a prolonged feeling of fullness and reducing food intake lead to significant weight loss (16). In addition, GLP-1- exerts neuroprotective as well as cardioprotective role by reducing inflammation, stimulating nerve growth and affecting lipid metabolism (11, 13).

Endogenous GLP-1 has a short half-life since it is in 1-2 minutes degraded by the enzyme dipeptidyl peptidase-4 (DPP-4). Further, active forms of GLP-1, as well as inactive metabolites, are rapidly eliminated by the kidneys (11, 13).

The short half-life of endogenous GLP-1 has led to the development of GLP-1 receptor agonists (GLP-1RAs) and DPP-4 inhibitors as therapeutic strategies to extend the duration of GLP-1 action in the treatment of type 2 diabetes (17).

## GLP-1 receptor agonists

The first approved GLP-1 receptor agonist (GLP-1 RA) was exenatide in the United States of America (USA) in 2005 and in Europe one year later. The exenatide dosing is inconvenient for the patient because it has to be injected twice daily to cover two major meals. In 2009, a second GLP-1 RA was approved – liraglutide, which has to be injected once daily. In the following years long acting, once weekly injected GLP-1 receptor agonists were approved (once-weekly exenatide, dulaglutide, albiglutide, semaglutide). In addition, first oral GLP-1 receptor agonist (oral semaglutide) was recently approved (18). Characteristic of approved GLP-1 receptor agonists are listed in **Table 1**.

**Table 1:** Characteristics of available GLP-1 receptor agonists.

GLP-1 receptor agonists	Administration route	Administration schedule	Elimination half-life	Side effects
Exenatide	Subcutaneous	Twice daily	3.3-4.0 h	Nausea, hypoglycemia, vomiting, diarrhea, feeling jittery, dizziness, headache, dyspepsia (19).
Liraglutide	Subcutaneous	Once daily	12.6-14.3 h	Nausea, vomiting, amylase and lipase level elevation, liver enzyme elevation, increase in gallbladder/biliary disease (20)
Lixisenatide	Subcutaneous	Once daily	2.6 h	Nausea, vomiting, headache, diarrhea, dizziness, hypoglycemia (21)
Once-weekly exenatide	Subcutaneous	Once weekly	3.3-4.0 h	Nausea, vomiting, diarrhea (22)
Dulaglutide	Subcutaneous	Once weekly	4.7-5.5 h	Nausea, vomiting, diarrhea (23)
Semaglutide	Subcutaneous	Once weekly	5.7-6.7 d	Nausea, vomiting, diarrhea, increased risk of cholelithiasis (24)
Semaglutide oral	Oral	Once daily	5.7-6.7 d	Nausea, abdominal pain, vomiting (25)
Albiglutide	Subcutaneous	Once weekly	5.7–6.8 d	Nausea, diarrhoea, and constipation, vomiting, injection site reaction (26)

**Figure 1:**
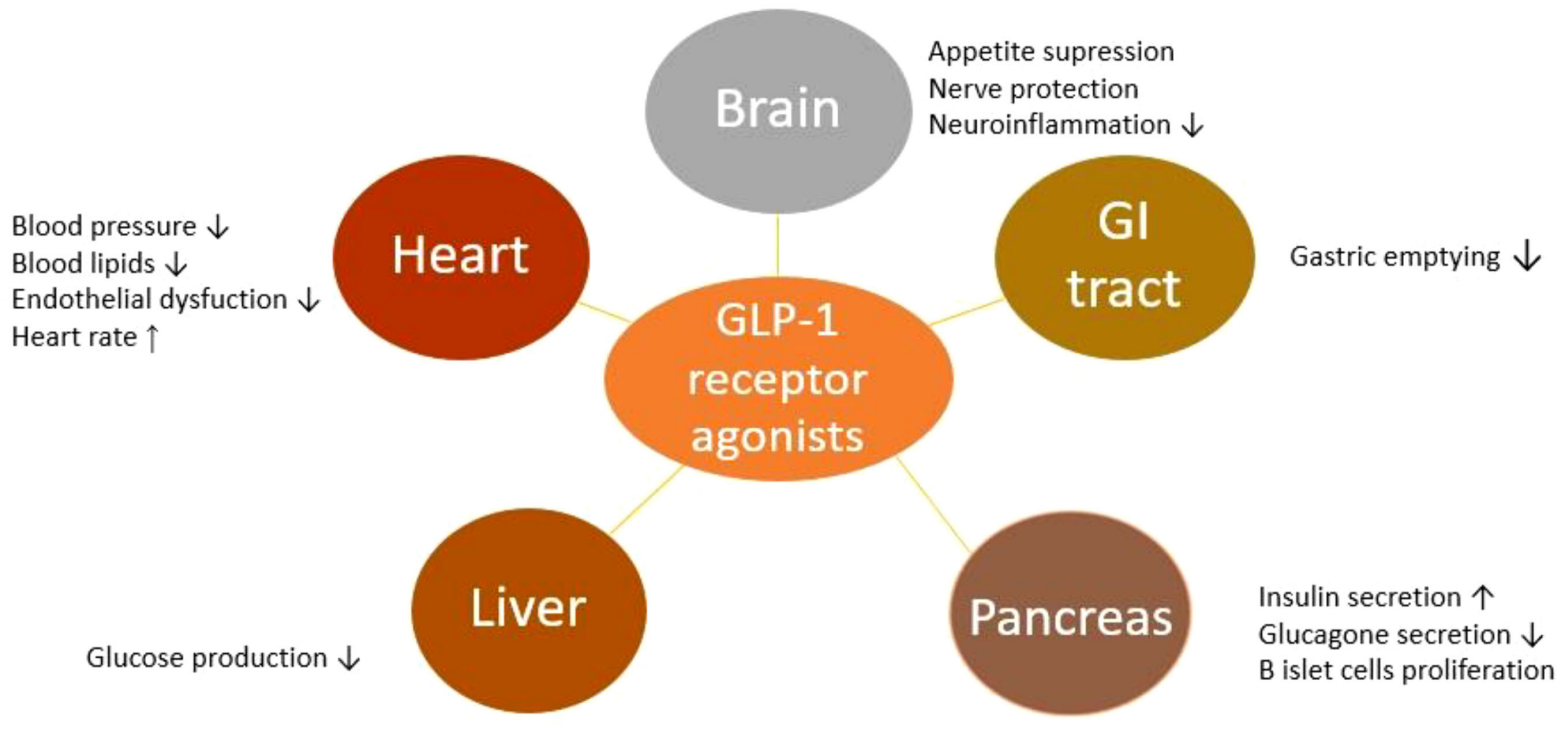
Positive effects of GLP-1 receptor agonists in humans.

GLP-1 receptor agonists augment insulin secretion after the meal and inhibit glucagon production from pancreatic alpha cells at hyper- or euglycemia. As well as endogenous GLP-1,GLP-1 receptor agonists also have numerous other effects such as promoting weight loss, deceleration of gastric emptying, lowering blood pressure (systolic and diastolic), and lowering total cholesterol (10, 27–29). Recently, it was confirmed that GLP-1 receptor agonists consistently reduce number of atherothrombotic events and have positive effects on kidney function in patients with type 2 diabetes (30). Positive effects of GLP-1 receptor agonist are shown in **Figure 1**.

The safety profile of GLP-1 receptor agonists has been evaluated in several meta-analyses. They consistently showed higher risk of mild to moderate gastrointestinal side effects in patients treated with GLP-1 receptor agonists. Meta-analysis of 34 trials showed that among GLP-1 receptor agonists, once-daily exenatide has the lowest risk of vomiting, while albiglutide had the highest risk for diarrhea. Another common side effect in GLP-1 RA treated patients was hypoglycemia, but with no difference in hypoglycemia rates between individual GLP-1 receptor agonists (31–33). When considering the GLP-1 receptor agonists as therapeutic option for patient, patient oriented approach should be used. Several factors should be considered such as magnitude of HbA1c reduction, effect on weight loss and adverse effect rate (34). The most common side effects of individual GLP-1 receptor agonists are listed in **Table 1**.

## Cardiovascular outcome trials

CVOTs have emerged as a pivotal component of assessing the impact of GLP-1 receptor agonists on cardiovascular health (35). Among the diverse array of antidiabetic agents, the class of GLP-1 receptor agonists has garnered substantial attention not only for their glycemic control properties but also for their potential cardiovascular benefits (8). CVOTs have become a cornerstone in evaluating antidiabetic medications, mandated by regulatory agencies to ensure that these drugs do not exacerbate cardiovascular risk. These trials are meticulously designed, randomized, placebo-controlled studies aimed at determine how antidiabetic medications influence cardiovascular outcomes, particularly in high-risk T2D patients (35). They serve as a critical bridge between diabetes management and cardiovascular health, addressing the pressing need to mitigate cardiovascular risk in this vulnerable patient population.

GLP-1 receptor agonists have demonstrated unique properties beyond glucose regulation. By mimicking the effects of endogenous GLP-1, they stimulate GLP-1 receptors, including those in cardiomyocytes and blood vessels (36). This dual action on both glycemic control and cardiovascular health has elevated GLP-1 receptor agonists to an important position in diabetes management. The profound influence of GLP-1 receptor agonists on cardiovascular outcomes has been elucidated through a series of CVOTs. The first goal of these trials was to prove GLP-1 receptor agonists cardiovascular safety, and majority of CVOTs were designed as non-inferiority trials. However, the results showed that in addition to the cardiovascular safety of GLP-1 receptor agonists, some drugs in the class can have influence on major adverse cardiovascular event (MACE) reduction compared to placebo (37). The accumulated evidence from CVOTs has not only informed clinical practice guidelines but has also empowered healthcare providers with a valuable therapeutic option for T2D patients who have high cardiovascular risk. Baseline characteristic and outcomes of CVOTs are listed in **Table 2**.

**Table 2:** Baseline characteristics of CVOTs involving GLP-1 receptor agonists and key findings.

	LEADER (n= 9340)	ELIXA (n= 6068)	SUSTAIN-6 (n=3297)	EXSCEL (n= 14752)	Harmony Outcomes (n=9463)	REWIND (n=9901)	PIONEER 6 (n= 3183)	AMPLITUDE-O (n=4076)
Drug	Liraglutide	Lixisenatide	Semaglutide	Exenatide	Albiglutide	Dulaglutide	Semaglutide	Efpeglenatide
Age, y	64 ± 7	60 ± 10	65 ± 7	62 ± 9	64 ± 7	66 ± 7	66 ± 7	65 ± 8
Sex - Male - Female	64% 36%	69% 31%	61% 39%	62% 38%	69% 31%	54% 46%	68% 32%	67% 33%
Administration route	Subcutaneous	Subcutaneous	Subcutaneous	Subcutaneous	Subcutaneous	Subcutaneous	Oral	Subcutaneous
Dosage	1.8 mg/d	10 µg/d or 20 µg/d	0·5 mg/w or 1 mg/w	2 mg/w	1,5 mg/w	1.5 mg/w	14 mg/d	4 mg/w or 6 mg/w
HbA1c %	8.7 ± 1.6	7.7 ± 1.3	8.7 ± 1.5	8.1 ± 1.0	8,7 ± 1.5	7.3 ± 1.1	8.2 ± 1.6	8.9 ± 1.5
Established CVD	81%	100%	83%	73%	100%	31%	85%	90%
History of HF	18%	22%	24%	16%	20%	9%	12%	18%
Follow up, y (median)	3.8	2.1	2.1	3.2	1.6	5.4	1.3	1.81
Key findings	Significantly reduced the risk of MACE	No significant impact on primary end-point	26% lower risk of MACE	Exenatide had no impact on primary end-point,	Significantly reduced the risk of MACE	Significantly reduced the risk of MACE	No significant reduction in MACE compared to placebo	Significantly lower risk of MACE and renal outcome events with
References	(37)	(38)	(39)	(40)	(41)	(42)	(43)	(44)

CV, cardiovascular; CVD, cardiovascular disease; HbA1c, hemoglobin A1c; HF, heart failure; MACE, major adverse cardiovascular events; mg, milligram; µg, microgram; w, week; y, year.

The LEADER (Liraglutide Effect and Action in Diabetes: Evaluation of Cardiovascular Outcome Results) trial was a pivotal milestone as it showed the cardiovascular benefits of liraglutide in patients with T2D and high cardiovascular risk. More than 9000 patients were included with follow up of 3.8 years. The results showed that in high risk patients with T2D liraglutide reduced the risk of MACE by 13% (primary endpoint included cardiovascular death, non-fatal myocardial infarction (MI), and non-fatal stroke) (37). The cardiovascular benefits of weekly injected GLP-1 receptor agonists have been proven in SUSTAIN-6 (Trial to Evaluate Cardiovascular and Other Long-term Outcomes with Semaglutide in Subjects with Type 2 Diabetes), REWIND (Researching Cardiovascular Events with a Weekly Incretin in Diabetes) and The Harmony outcomes trials. Semaglutide in SUSTAIN-6 trial reduced the risk of MACE by 26% in patients with T2D, mostly by reducing rate of nonfatal stroke (39%) compared to placebo. Similar results were seen in the REWIND study were dulaglutide also reduced the risk of nonfatal strokes compared to placebo. In both trials, there was no difference in rates of nonfatal myocardial infarction and cardiovascular death between GLP-1 RA treated patients and placebo. While SUSTAIN-6 and REWIND trials examined GLP-1 RA in diabetics with different cardiovascular risk profile, The Harmony Outcomes study enrolled patients with T2D and established atherosclerotic cardiovascular disease (ASCVD). In those high risk patients, albiglutide significantly reduced MACE compared to placebo, and unlike semaglutide in SUSTAIN-6 trial this difference was most prominent in reducing the rate of myocardial infarction (39, 41, 42). Efpeglenatide, another once weekly injected GLP-1 receptor agonist, was evaluated in AMPLITUDE-O trial. The AMPLITUDE-O trial included 4076 high risk patients (patients with established cardiovascular or kidney disease and at least one cardiovascular risk factor) and it showed that efpeglenatide in those high risk patients reduces MACE and worsening kidney function (44). In the aforementioned trials, except the REWID trial, the most represented patients were those with established cardiovascular diseases, indicating that efficacy of GLP-1 receptor agonists is proven in terms of secondary prevention. The REWIND trial included only 31% patients with established cardiovascular disease, thereby it provided evidence for dulaglutide efficacy in primary prevention.

In the ELIXA and the EXSCEL trials, GLP-1RAs (once daily lixisenatide and once weekly exenatide, respectively) didn’t provide benefit in MACE reduction compared to placebo, but were non-inferior to placebo for the primary composite outcome of MACE. ELIXA trial included patients with recent cardiovascular event (patients with acute coronary syndrome 180 days before randomization), and although there was no benefit in MACE reduction in lixisenatide treated patients compared to placebo, results revealed cardiovascular safety of lixisenatide in this specific high risk population (38, 40). An innovative oral form of GLP-1 receptor agonist was assessed in The Peptide Innovation for Early Diabetes Treatment (PIONEER) 6 trial. Published in 2019, it did not show superiority in reducing MACE, but it provided valuable evidence regarding the safety and oral delivery of GLP-1 receptor agonists, expanding treatment options for patients with T2D (43). Cardiovascular benefit of oral semaglutide in patients with T2DM and established atherosclerotic cardiovascular diseases and/or chronic kidney disease is currently under investigation in SOUL (Semaglutide cardiovascular outcomes) trial (45). Meta-analysis of these eight CVOTs with 60 080 patients included showed that GLP-1 receptor agonists reduce MACE and have a positive impact on chronic kidney disease in patients with T2D (30).

Dose-dependent effect for oral semaglutide was assessed and proven in Japanese PIONEER 9 trial, where greater reduction of HbA1c and weight loss was achieved with higher dose of oral semaglutide compared to placebo and 0.9 mg liraglutide (46). Similar effect was obtained with escalation of dulaglutide from 1.5 mg to 3 mg or 4.5 mg; HbA1c and weight loss reduction were dose-related with similar safety profile between higher and lower dose (47). In AMPLITUDE-O possible dose-dependent effect for the MACE occurrence was observed. Estimated hazard ratio for 6 mg/weekly dose of efpeglenatide compared to placebo was 0,65 (95% CI, 0.5 to 0.86) and 0.82 (95% CI 0.63 – 1.06) for 4 mg/weekly dose compared to placebo (44). In SUSTAIN-6 trial similar effects in MACE risk reduction, glycemic control and hypoglycemia rates were observed in patients receiving 0.5 mg semaglutide once weekly and in those receiving 1 mg once weekly (43). In other CVOTs most patients received target dose (maximal tolerated dose) and no analysis was done regarding dose-dependent effects. These CVOTs have collectively contributed significantly to our understanding of the cardiovascular outcomes related to GLP-1 receptor agonists. These trials have enriched the therapeutic landscape for T2D patients, offering a range of GLP-1 receptor agonists with varying cardiovascular profiles, thus allowing clinicians to personalize treatment for each patient.

## Mechanisms related to cardiovascular effects of GLP-1 receptor agonists

The cardiovascular effects of GLP-1 receptor agonists in CVOTs can be attributed to several mechanisms. Understanding the mechanisms underlying these cardiovascular benefits is crucial for optimizing the treatment of T2D. While glycemic control is not traditionally considered a direct cardiovascular risk factor, it is pivotal in mitigating cardiovascular risk in T2D patients. GLP-1 receptor agonists act primarily by enhancing postprandial insulin secretion and suppressing glucagon release, leading to improved glycemic control (8). Tight glucose management, as demonstrated in landmark clinical trials such as the Diabetes Control and Complications Trial (DCCT) and the United Kingdom Prospective Diabetes Study (UKPDS), has been related to reduced risk of cardiovascular events. Maintaining stable blood glucose levels through lifestyle modifications and medications can mitigate the adverse effects of hyperglycemia on blood vessels, thereby contributing to CVD risk reduction in diabetic patients (48, 49).

Emerging evidence suggests that GLP-1 receptors are not confined to pancreatic islets but are also expressed in cardiomyocytes and vascular endothelial cells (50, 51). GLP-1 receptor agonists promote myocardial glucose uptake and utilization, reduce oxidative stress, and inhibit cardiomyocyte apoptosis (52). These mechanisms collectively have cardioprotective effects on heart function and prevention of adverse cardiac remodeling.

GLP-1 receptor activation induces vasodilation through various pathways. It stimulates production of endothelial nitric oxide (NO), a potent vasodilator, leading to the relaxation of blood vessels and improved coronary blood flow (53, 54). Additionally, GLP-1 agonists may modulate the renin-angiotensin-aldosterone system (RAAS), further influencing vascular tone and blood pressure regulation (54). The vasodilatatory effects of GLP-1 agonists contribute to reduced systemic vascular resistance and, consequently, lower blood pressure. Another mechanism by which GLP1 receptor agonists lower blood pressure is through enhancing natriuresis (55).

Numerous studies have proven that GLP-1 receptor agonists consistently lead to weight loss reducing appetite, enhancing fullness, slowing gastric emptying, and reducing food intake. Weight reduction has many positive effects, from improving insulin sensitivity to reducing the risk of cardiovascular diseases (40, 56, 57). Meta-analysis of 35 clinical trials demonstrated a modest reduction in LDL cholesterol, total cholesterol, and triglycerides in patients treated with GLP-1 RAs compared to controls (4). In addition to above mentioned properties, GLP-1 RAs have an antiinflammatory effect; in comparison with other antidiabetic treatments GLP-1 RAs have significantly reduced inflammatory biomarkers (58). These antiinflammatory and antiatherogenic effects of GLP-1 RAs reduce formation of atherosclerotic lesions and progression (59, 60).

The effects of GLP-1 receptor agonists on cardiovascular system extend beyond their primary role in glycemic control. These agents act through multiple mechanisms, including direct cardioprotection, vasodilation, natriuresis, weight reduction, lipid profile improvement, anti-inflammatory effects, renal protection, reduction in plaque formation, and neurohormonal regulation. These findings underscore the multifaceted nature of GLP-1 receptor agonists in cardiovascular risk reduction and have profound implications for the treatment of different profiles of T2D patients.

## GLP-1 receptor agonists and heart failure

Heart failure (HF) is a complex and debilitating condition with a substantial global health burden (61). In recent years, GLP-1 receptor agonists, have garnered significant attention for their potential benefits in heart failure management. However, clinical trials so far have not shown clear benefit of GLP-1 RAs in HF. The influence of GLP-1 receptor agonists on heart failure with reduced ejection fraction has been investigated so far only by two small clinical trials, the FIGHT and the LIVE trial. In both trials study drug was liraglutide. The FIGHT trial included 300 patients with acute heart failure and reduced ejection fraction (< 40%); there was no observed benefit in liraglutide treated patients compared to placebo in primary outcome (62). The LIVE trial assessed the influence of liraglutide in patients with chronic HF and reduced left ventricular ejection fraction (LVEF ≤45%). In this trial, liragutide treated patients not only did not benefit from the therapy, but also had a higher risk of developing arrhythmia and acute coronary syndrome compared to placebo (63). Another small trial, the Effect of Semaglutide 2.4 mg Once Weekly on Function and Symptoms in Subjects with Obesity-related Heart Failure with Preserved Ejection Fraction (STEP-HFpEF) trial assesed semaglutide efficay in patients with preserved LVEF (LVEF ≥ 45%) and obesity (BMI ≥ 30 kg/m^2^). In this trial semaglutide led to greater weight loss, heart failure related symptomes improvement and to significant difference in 6-minute walk distance when compared to placebo. The significance of STEP-HFpEF trial is that it demonstrated benefits of semaglutide in nondiabetic patients with HFpEF and obesity. The effect of 2.4 mg once-weekly injected semaglutide in patients with HFpEF, obesity and T2D is currently investigated in an ongoing trial STEP-HFpEF DM (Semaglutide Treatment Effect in People with obesity and HFpEF and type 2 diabetes) (64, 65).

In eight CVOTs history of heart failure was reported at baseline and it ranged from 9% to 24% with no significant difference between patients receivng GLP-1 receptor agonist and patients receiving placebo. Heart failure was not clearly defined in trials protocols, left ventricular ejection fraction was reported only in EXSCEL trial and in 4 trials heart failure functional class was defined. NYHA class IV was exclusion criteria in ELIXA, LEADER and SUSTAIN-6 trials. Results failed to show the benefit of GLP-1 receptor agonists in reducing the risk of heart failure; in the LEADER, REWIND, EXSCEL, ELIXA and SUSTAIN-6 trials were no significant difference in HF hospitalization between GLP-1 receptor agonist and placebo group (37–44, 66). Results for albiglutide were also neutral in the Harmony Outcomes Study, although hospitalization for heart failure was assesed composite with cardiovascular death as a secondary endpoint (41). No benefit in terms of the risk of HF hospitalization was observed with oral semaglutide, as well as with efpeglenatide (43, 44). Analysis of subgroup of patients with HF in the EXSCEL trial revelaed that use of once-weekly exenatide reduced the risk of all-cause mortality in patient without HF (HR 0.79 [95% CI, 0.68–0.92]), while in patients with baseline HF no effect was observed (HR, 1.05 [95% CI, 0.85–1.29) (67).

However, meta-analysis encompassing eight CVOTs showed positive impact of GLP-1 receptor agonists on heart failure in patients with type 2 diabetes (T2D). Meta-analysis of these 8 CVOTs included more than 60 000 patients and aimed to evaluate the influence of GLP-1 receptor agonists on cardiovascular system. The findings from this meta-analysis revealed promising trends regarding heart failure outcomes. Results confirmed positive impact of GLP-1 receptor agonists on MACE outcomes in patients with T2D but also revealed promising trends regarding heart failure outcomes in terms of reduced rate of heart failure-related events (30).

In conclusion, GLP-1 receptor agonists show potential in heart failure management through their multiple mechanisms of action, including direct cardioprotective effects, vasodilation, natriuresis, and glucose and weight control. While clinical trials have provided valuable insights, further research is should determine the optimal use of these agents in different heart failure populations.

## GLP-1 receptor agonists, SGLT2 inhibitors and DPP-4 inhibitors

GLP-1 receptor agonists, sodium glucose co-transporter-2 (SGLT2) inhibitors and dipeptidyl peptidase-4 (DPP-4) inhibitors reduce the risk of MACE compared to placebo. Recently published four-arm target trial which included 283 998 patients compared effectiveness of SGLT2 inhibitors, GLP-1 receptor agonists, DPP-4 inhibitors and sulfonylureas. Results showed that GLP-1 receptor agonists and SGLT2 inhibitors were superior in reducing the risk of MACE when compared to DPP-4 inhibitors (HR 0·86 [0·82–0·89] and 0·86 [0·82–0·90], respectively), but no difference was found between SGLT2 inhibitors and GLP-1 receptor agonists in reducing rate of MACE (HR of 1·01 (0·94–1·07)) (68). Similar results were obtained in meta-analysis of twenty-three trials encompassing 181 143 patients. As in the previously mentioned study there was no difference between GLP-1 receptor agonists and SGLT2 inhibitors in reducing the risk of MACE, but when compared to DPP-4 inhibitors SGLT2 inhibitors significantly reduced cardiovascular and total death; and GLP-1 receptor agonists significantly reduced total death. In the same meta-analysis SGLT-2 were superior to GLP-1 receptor agonists in terms of reducing hospitalization for HF and the composite renal outcome, while GLP-1 receptor agonists were the only drug that reduced non-fatal stroke (69). Trials so far have shown superiority of GLP-1 receptor agonists and SGLT2 inhibitors in MACE reduction when compared to DPP-4 inhibitors. Both, SGLT2 inhibitors and GLP1 receptor agonists, had similar effect on MACE reduction, but SGLT2 inhibitors have far more benefits in patients with heart failure.

## Conclusion

In conclusion, CVOTs have played a pivotal role in evaluating the cardiovascular safety and efficacy of GLP-1 receptor agonists in patients with T2D. The results of these trials have consistently demonstrated the cardiovascular benefits associated with GLP-1 receptor agonists including reductions in major adverse cardiovascular events. These findings have provided valuable insights into the management of T2D and informed clinical practice guidelines and regulatory decisions.GLP-1 receptor agonists have emerged as an important therapeutic option for individuals with T2D, particularly those at high risk of CVD. Their multiple effects beyond glycemic control makes them a valuable addition to the armamentarium of antidiabetic medications. As research in this field continues to evolve, ongoing and future CVOTs will likely expand our knowledge of the cardiovascular benefits of GLP-1 receptor agonists, ultimately improving the care and outcomes of individuals with T2D and CVD.

## Author contributions

LF: Conceptualization, Methodology, Project administration, Writing – original draft. DM: Conceptualization, Methodology, Writing – review & editing. AM: Formal Analysis, Methodology, Writing – review & editing.
